# Vitamin D activates FBP1 to block the Warburg effect and modulate blast metabolism in acute myeloid leukemia

**DOI:** 10.1186/s40364-022-00367-3

**Published:** 2022-04-02

**Authors:** Yi Xu, Christopher Hino, David J. Baylink, Jeffrey Xiao, Mark E. Reeves, Jiang F. Zhong, Saied Mirshahidi, Huynh Cao

**Affiliations:** 1 Division of Hematology and Oncology, Department of Medicine, Loma Linda University, Loma Linda, CA USA; 2 Division of Regenerative Medicine, Department of Medicine, Loma Linda University, Loma Linda, CA USA; 3 Loma Linda University Medical Center & Loma Linda University Cancer Center, 11234 Anderson Street, Room 1524, Loma Linda, CA 92354 USA; 4 Department of Basic Sciences, Loma Linda University, Loma Linda, CA USA; 5 Biospecimen Laboratory, Loma Linda University Cancer Center, Department of Medicine and Basic Sciences, Loma Linda University School of Medicine, Loma Linda, CA 92354 USA

**Keywords:** AML, Vitamin D, FBP1, Metabolism, Warburg effect, Glycolysis

## Abstract

Acute myeloid leukemia (AML) has the lowest survival rate among the leukemias. Targeting intracellular metabolism and energy production in leukemic cells can be a promising therapeutic strategy for AML. Recently, we presented the successful use of vitamin D (1,25VD3) gene therapy to treat AML mouse models in vivo*.* In this study, recognizing the importance of 1,25VD3 as one of only 2 molecules (along with glucose) photosynthesized for energy during the beginning stage of life on this planet, we explored the functional role of 1,25VD3 in AML metabolism.

Transcriptome database (RNA-seq) of four different AML cell lines revealed 17,757 genes responding to 1,25VD3-treatment. Moreover, we discovered that fructose-bisphosphatase 1 (FBP1) noticeably stands out as the only gene (out of 17,757 genes) with a 250-fold increase in gene expression, which is known to encode the key rate-limiting gluconeogenic enzyme fructose-1,6-bisphosphatase. The significant increased expression of FBP1 gene and proteins induced by 1,25VD3 was confirmed by qPCR, western blot, flow cytometry, immunocytochemistry and functional lactate assay. Additionally, 1,25VD3 was found to regulate different AML metabolic processes including gluconeogenesis, glycolysis, TCA, de novo nucleotide synthesis, etc. In summary, we provided the first evidence that 1,25 VD3-induced FBP1 overexpression might be a novel therapeutic target to block the “Warburg Effect” to reduce energy production in AML blasts.

**Supplementary Information:**

The online version contains supplementary material available at 10.1186/s40364-022-00367-3.

To the Editor,

Acute myeloid leukemia (AML) is the most common type of leukemia in adults [1]. Despite improvements in our understanding of AML and the development of different therapeutic approaches, approximately 50% of patients will relapse following induction chemotherapy, resulting in a dismal 5-year overall survival rate of 29% [1, 2]. As such, there is an unmet need to understand the fundamental mechanisms of relapsed/refractory AML and develop effective therapies to improve the prognosis of AML. The remodeling of cellular metabolism is an essential process to meet higher demands of energy in cancers [3]. Enhanced glycolysis, known as the “Warburg Effect,” has been confirmed in leukemic blasts and is correlated to a worse prognosis for AML [4]. Also, increased production of lactate was attributed to chemoresistance in AML patients who have up-regulated lactate dehydrogenase [5]. Therefore, identifying potential druggable targets in a complex network of metabolic processes and developing relevant treatment approaches to inhibit blast metabolism/energy production could be one promising therapeutic strategy for AML/its relapse [6]. Fructose-1,6-bisphosphatase (FBP1) is an essential enzyme for gluconeogenesis, the pathway that runs opposite of glycolysis by transforming substrates into glucose, and based on prior studies of different solid tumors, FBP1 can also function as a tumor suppressor by inhibiting glycolysis and cancer cell growth [7].

Vitamin D is known to be the oldest hormone on earth [8]. Some of the earliest life forms such as phytoplankton took advantage of sunlight to photosynthesize 2 metabolites for energy and survival: glucose and vitamin D [8]. Our recent study demonstrated that the combination of 1,25VD3 and 5-Azacytidine (a FDA-approved hypomethylating agent) enhanced cytotoxicity/differentiation and inhibited proliferation of AML blasts in vivo [9]. Up to 35% of AML patients have mutations in the FMS-like receptor tyrosine kinase 3 (FLT3) gene and defective protein products (AML-FLT3) that are associated with poorer survival through an increased risk of relapse [10]. Tyrosine kinase inhibitors (TKI) are a new type of targeted therapies that are in clinical trials for the treatment of AML-FLT3 patients [11]. Our preliminary in vitro studies revealed that the supplementation of 1,25VD3 to Midostaurin (MIDO), a 1st generation TKI could effectively suppress the proliferation of MV4–11 (Supple. Fig. 1A). Our qPCR data also confirmed that the combination of 1,25VD3 and Gilteritinib (GILT), a 2nd generation TKI, could significantly reduce the CYCLIN D1 (encoded by CCND1 gene, 93% downregulation versus the untreated control and superior to single agents, Supple. Fig. 1B). This data is consistent with previous findings showing 1,25VD3 controls G1-S phase-cycle machinery in human breast cancer cells by repressing the CCND1 gene [12]. A recent study suggests that numerous metabolic pathways except for gluconeogenesis can be therapeutically exploited to overcome the TKI-resistance [13], and inhibition of glutaminolysis can achieve a promising treatment effect on AML-FLT3 blasts [14]. Vitamin D supplementation has been also found to correct the metabolic disturbance caused by a fructose-rich diet [15]. In this study, to find new therapeutic targets and develop potential 1,25VD3-based treatments for AML, we explored the comprehensive details of how 1,25VD3 works on the metabolism of FLT3-mutated blasts.

First, we performed transcriptome analyses (RNA-seq) of 4 different AML-FLT3 cell lines including MV4–11, MOLM-14, MV4–11-midostaurin-resistant cells and MOLM-14-midostaurin-resistant cells which were previously reported [9]. Among many differential expression methods developed for RNA-seq data analyses, FPKM number was found to be one of the best approaches in precision and accuracy on reporting RNA-seq results [16]. Our RNA-seq database revealed that there were 17,757 genes with FPKM numbers after 1,25VD3 treatment (distribution pie, Fig. 1A). FBP1 was found as the only gene with a ~ 254-fold increase in gene expression, which was ranked at 8413th (4.37 FPKM) in the untreated group and then at the 94th rank (1110.13 FPKM) after 80 nM 1,25VD3 treatment among 17,757 genes analyzed (Fig. 1B). Similar changes in FPKM and ranks could be observed in all 4 cell lines (Fig. 1B). The significantly increased FBP1 gene and proteins were confirmed by immunocytochemistry, qPCR and western blot (Fig. 1C-F). Furthermore, the functional lactate assay showed the significant reduction of the lactate concentration in MV4–11 cells after 1,25VD3 treatment (Fig. 1G). The flow cytometry (FC) data showed that 95.9% of the FBP1+ cells expressed vitamin D receptor (VDR, Fig. 1D; Isotype control in Supple. Fig. 2). In addition to MV4–11/MOLM-14, significant elevation of FBP1 and induction of blast differentiation could be observed in 1,25VD3 treatment of HL60, a human acute promyelocytic leukemia (APL) cell line (Supple. Fig. 3). The detailed description of materials and methods is available in supplementary documents.

**Fig. 1:**
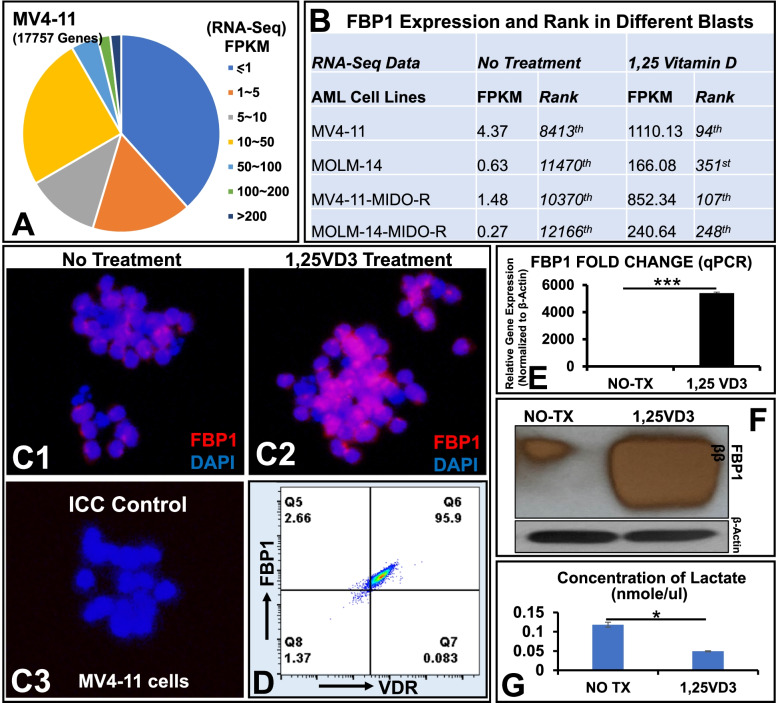
1,25 vitamin D induced FBP1 expression and reduced lactate production. **A** Pie distribution of RNA-seq FPKM-based gene expression in MV4–11 cells; **B** The FBP1 expression (FPKM) increased sharply from the low rank in non-treated (NO-TX) group to the high rank in 80 nM 1,25VD3-treated groups in different experimental groups of MV4–11, MOLM-14 cells, MV4–11-MIDO-R and MOLM-14-MIDO-R cells (MV4–11 or MOLM-14 resistant to midostaurin). MIDO: midostaurin (80 nM); **C**1–3 40x Images from Immunocytochemistry (ICC) to compare FBP1 protein before or after 1,25VD3 treatment; ICC control: 2nd antibody was applied without the primary antibody; **D** Representative FC plots showing the co-expression of FBP1 and VDR in 1,25VD3-treated MV4–11 cells; The Supplementary Figure 2 showing the FC plot of FITC-isotype control; **E** MV4–11 cells were treated with 80 nM 1,25VD3 for 48 h, then harvested and analyzed by RT-qPCR for expression of human FBP1 (Fold Change); **F** Treated MV4–11 cells were analyzed by WB for protein expression of human FBP1; **G** Treated MV4–11 cells were analyzed by Lactate Assay; Cumulative data of the concentration of intracellular lactate; Where applicable, data are means ± SEM and were analyzed by student “t” test. *p < 0.05, ***p < 0.005, *n* = 5

In addition to the central pathway of metabolizing glucose to pyruvate via glycolysis, AML metabolism involves diverse processes of nucleotides, amino acid, lipids and their end metabolites to perform signaling functions and produce energy to support tumorigenesis [17]. Here, we provided a table of RNA-seq data showing how 1,25VD3 modulated the genes essential for different metabolite processes in both MV4–11 and MV4–11-MIDO-R cells (Fig. 2A). Notably, 1,25VD3 was found to increase gene expressions of certain enzymes related to gluconeogenesis, TCA cycles, oxidative phosphorylation, glycogenesis, and reduce gene expressions of certain enzymes related to glycolysis, glycogenolysis and nucleotide synthesis (Fig. 2A). In summary, our report is the first to identify the pathway of vitamin D modulating the AML metabolism by activating FBP1 to block the “Warburg Effect”, which might enhance its anti-leukemic effect in addition to the induction of differentiation and inhibition of cell cycle progression (Fig. 2B). However, in prior clinical trials of vitamin D treatments for AML, there have been mixed results: this is probably due to the varying expression of baseline VDR of leukemic blasts and loss of function in mutated VDR [18]. The significant 1,25VD3-induced up-regulation of FBP1 to suppress glycolysis and its co-expression with VDR provides an important clinical implication that FBP1 could be a novel therapeutic target for the treatment of AML/its relapse by bypassing the impaired or low baseline VDR expression.

**Fig. 2:**
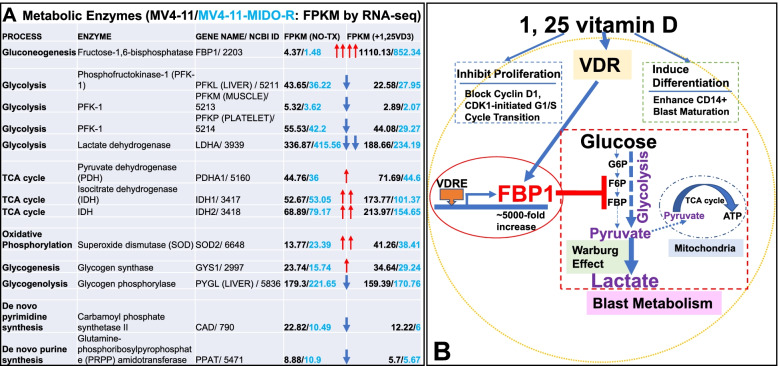
1,25 vitamin D activates FBP1 to modulate AML Metabolism and block the “Warburg Effect” to enhance its anti-leukemic effect. **A** Table of RNA-seq results revealing that 1,25VD3 (80 nM) modulated different metabolic pathways in MV4–11 and MV4–11-MIDO-R cells by increasing gene expressions of certain enzymes related to gluconeogenesis, TCA cycles, oxidative phosphorylation, glycogenesis, and reducing gene expressions of certain enzymes related to glycolysis, glycogenolysis and nucleotide synthesis. **B** A summarized diagram. In addition to 1,25VD3’s known roles in inducing differentiation and inhibiting proliferation, we proposed a new functional role of vitamin D in the treatment of AML blasts. 1,25VD3 induces ~ 5000-fold increase of FBP1 (qPCR data) in AML blasts, which encodes large amounts of Fructose-1,6-bisphosphatase (extremely large bands in WB) to disrupt the progression of glycolysis and reduce the lactate production (Warburg Effect), a main energy resource for AML metabolism

## Supplementary Information

**Additional file 1.**

## Data Availability

The datasets used and/or analyzed in the current study are available from the corresponding author.
