# Correction: Vitamin D activates FBP1 to block the Warburg effect and modulate blast metabolism in acute myeloid leukemia

**DOI:** 10.1186/s40364-022-00379-z

**Published:** 2022-05-19

**Authors:** Yi Xu, Christopher Hino, David J. Baylink, Jeffrey Xiao, Mark E. Reeves, Jiang F. Zhong, Saied Mirshahidi, Huynh Cao

**Affiliations:** 1 Division of Hematology and Oncology, Department of Medicine, Loma Linda University, Loma Linda, CA USA; 2 Division of Regenerative Medicine, Department of Medicine, Loma Linda University, Loma Linda, CA USA; 3 Loma Linda University Medical Center & Loma Linda University Cancer Center, 11234 Anderson Street, Room 1524, Loma Linda, CA 92354 USA; 4 Department of Basic Sciences, Loma Linda University, Loma Linda, CA USA; 5 Biospecimen Laboratory, Loma Linda University Cancer Center, Department of Medicine and Basic Sciences, Loma Linda University School of Medicine, Loma Linda, CA 92354 USA

**Correction: Biomarker Res 10, 1-5 (2022)**

**https://doi.org/10.1186/s40364-022-00367-3**

The original article [1] contained a typographical error in Fig 1F as well as some revision highlights in the supplementary material. These errors have since been corrected.

**Fig. 1:**
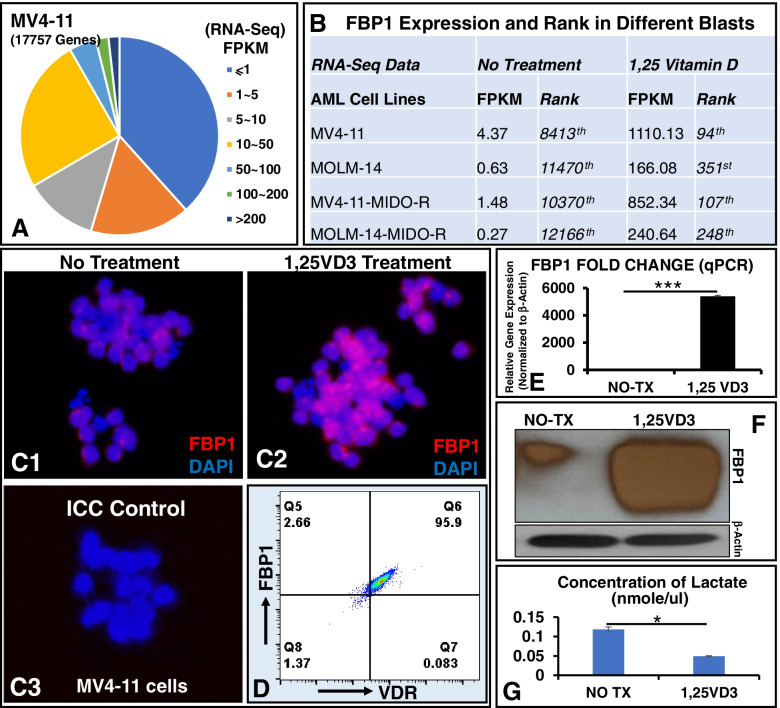
1,25 vitamin D induced FBP1 expression and reduced lactate production. **A** Pie distribution of RNA-seq FPKM-based gene expression in MV4–11 cells; **B** The FBP1 expression (FPKM) increased sharply from the low rank in non-treated (NO-TX) group to the high rank in 80 nM 1,25VD3-treated groups in different experimental groups of MV4–11, MOLM-14 cells, MV4–11-MIDO-R and MOLM-14-MIDO-R cells (MV4–11 or MOLM-14 resistant to midostaurin). MIDO: midostaurin (80 nM); **C**1–3 40x Images from Immunocytochemistry (ICC) to compare FBP1 protein before or after 1,25VD3 treatment; ICC control: 2nd antibody was applied without the primary antibody; **D** Representative FC plots showing the co-expression of FBP1 and VDR in 1,25VD3-treated MV4–11 cells; The Supplementary Figure 2 showing the FC plot of FITC-isotype control; **E** MV4–11 cells were treated with 80 nM 1,25VD3 for 48 h, then harvested and analyzed by RT-qPCR for expression of human FBP1 (Fold Change); **F** Treated MV4–11 cells were analyzed by WB for protein expression of human FBP1; **G** Treated MV4–11 cells were analyzed by Lactate Assay; Cumulative data of the concentration of intracellular lactate; Where applicable, data are means ± SEM and were analyzed by student “t” test. *p < 0.05, ***p < 0.005, *n* = 5

## Supplementary Information

**Additional file 1.**
