# Supplementary material for: Correction: Vitamin D activates FBP1 to block the Warburg effect and modulate blast metabolism in acute myeloid leukemia
Source: Biomark Res. 2022 May 19;10:33. doi: 10.1186/s40364-022-00379-z (PMC9121558; doi:10.1186/s40364-022-00379-z)
Supplement: Supplementary file 1 — Additional file 1. [file 40364_2022_379_MOESM1_ESM.docx]

**Vitamin D Activates FBP1 To Block the Warburg Effect and Modulate Blast Metabolism in Acute Myeloid Leukemia**

Yi Xu^1, 2, 3@^, Christopher Hino^1^, David J. Baylink^2^, Jeffrey Xiao^2^, Mark E. Reeves^1, 3^, Jiang F. Zhong^4^, Saied Mirshahidi^5^, Huynh Cao^1,3^

**Materials and Methods**

The list of reagents including manufacturers and catalogues of antibodies and kits are found in the supplementary data (**Supplementary** **Table 1**).

**Cell Culture of AML cells and Treatment of blasts by active vitamin D (1,25VD3 or 1,25(OH)_2_D_3_) and TKIs**

MV4-11 (ATCC CRL-9591) and MOLM-14 (DSMZ ACC-777) are human-derived AML blast cell lines with FLT3 mutation. The generation of MOLM-14-TKI-resistent and MV4-11-TKI-resistant cell lines were previously reported [1]. The AML cells were cultured in RPMI-1640 medium (Hyclone) supplemented with 10% heat-inactivated fetal bovine serum (HyClone) and 1% penicillin/streptomycin. Cells were grown at 37ﹾC in a humidiﬁed atmosphere containing 5% CO2. As single agents to treat blasts *in vitro*, one dose of 80nM of 1,25VD3 was added to 1 ml of 1x 10^6^ MV4-11 cells for each experimental group in 24 well plates. Two days after the treatment, cells were then collected for further analyses of gene or protein expressions.

**RNA-sequencing and data processing**

AML cells from MV4-11, MOLM-14, MV4-11-TKI-resistant and MOLM-14-TKI-resistent cell

lines were treated for 48 hours with one dose of 80 nM 1,25VD3, 80 nM Midostaurin (MIDO) or

a combination of 80 nM 1,25VD3 and 80 nM MIDO. Cell samples were collected and sent to BGI

for RNA sequencing (RNA-seq). The RNA-seq libraries were prepared and fragments per kilobase

of exon per million fragments mapped (FPKM) were applied to compare gene expression of

samples between different treatment groups (**Fig.1**). Among many differential expression methods

develpoed for RNA-seq data analyses, FPKM number was found to one of the best approaches in

precision and accuracy to report RNA-seq results [2].

**Flow Cytometry (FC)**

Cells were harvested and examined for the expression of cell surface biomarkers and intracellular proteins by multichromatic FC as previously described [3]. About 1 x 10^4^ ~ 10^6^ cells in 100 µl FC buffer (PBS containing 1% FBS and 0.05% sodium azide) were stained with various fluorescence-conjugated antibodies specific for the desired cell surface proteins at 4^o^C for 30 minutes. The surface-stained cells were then fixed and permeabilized using the appropriate reagents (e.g. the BD Pharmingen Cytofix/Cytoperm buffer) and stained with different fluorescence-conjugated antibodies specific for the desired intracellular proteins at 4^o^C for 2 hours in the permeabilizing buffer (e.g. the BD Perm/Wash buffer). Concentrations of the Abs were used per the manufacturers’ recommendations (**Supplementary** **Table 1**). Finally, the cells were washed twice in the permeabilizing buffer and twice in the FC buffer before being analyzed on the BD FACSAria II. Data was analyzed using the FlowJo software (Tree Star Inc., Ashland, OR).

**RNA isolation and** **Real-Time Polymerase Chain Reaction (qPCR) analysis**

MV4-11 was cultured with the presence of appropriate treatments for 48 hours. Cells were collected for RNA isolation and qPCR analysis as previously described [1]. Total RNA was isolated using the RNeasy Micro Kit (Qiagen) according to the manufacturer’s instruction. First-strand cDNA was synthesized using the SuperScript III Reverse Transcriptase (Invitrogen). With an Applied Biosystems 7900HT Real-Time PCR machine, qPCR was performed and analyzed. The primer sequences of FBP1 are GCAGTCAAAGCCATCTCTTCGG and TAACCAGGTCGTTGGAGAGGAC. The primer sequences of β-actin are CACCATTGGCAATGAGCGGTTC and AGGTCTTTGCGGATGTCCACGT. The PCR conditions were 10 minutes at 95°C followed by 40 cycles of 10 seconds at 95°C and 15 seconds at 60°C. The relative expression level of a gene was determined using the ΔΔCt method and normalized to β-Actin.

**Western Blotting Analysis**

AML cells were homogenized in ice-cold lysis buffer composed of 20mM Hepes, pH 7.5, 10mM KCl, 1.5mM MgCl2, 1mM ethylenediaminetetraacetic acid, 1mM dithiothreitol, 1mM phenylmethylsulfonyl fluoride, 2 µg/ml of aprotinin, and 10 µg/ml of leupeptin, followed by incubation on ice for 30 minutes. The homogenates were ultrasonicated and centrifuged at 20000 X g for 30 minutes at 4ºC. Samples with equal quantities of protein were loaded onto 10% SDS-polyacrylamide gel and separated by electrophoresis at 100 V for 1 hour. Proteins were then transferred onto Immobilon-P membranes (Millipore Corporation, Billerica, MA) and were probed with the primary antibody of FBP1 (Invitrogen), and then incubation with horseradish peroxidase-conjugated secondary antibody (Amersham, Arlington Heights, IL). Because 1,25VD3-induced FBP1 proteins are too strong, we had to modify the developing process. Briefly, FBP1 proteins were visualized with a chemiluminescence reagent in the dark room, and immediately the blots were exposed to double or triple-stacked films for 1 second (Hyperfilm, Amersham). The exposed films were then developed within minutes, and results were quantified using the Kodak electrophoresis documentation and analysis system and Kodak ID image analysis software (Eastman Kodak, Rochester, NY).

**Immunocytochemistry (ICC) and imaging Acquisition**

ICC staining of treated MV4-11 cells was performed according to the established protocol in the previous report [4]. Fluorescent images were taken using an Olympus 1X71 microscope and were processed using an Olympus cellSens Dimension 1.15 Imaging Software.

**Lactate Assay**

MV4-11 cells were treated with or without 1,25VD3 for 48 hours. The cell number of each experimental group was counted to have equal cell numbers per sample. Then, the cell samples were processed according to the manufacturer’s protocol (Catalog Number: MAK064, Sigma-Aldrich). Briefly, the lactate concentration of each sample was determined by an enzymatic assay, resulting in a colorimetric product, proportional to the lactate present. The experimental plate was read with a spectrophotometric microplate reader at 570 nm. The concentration of lactate within samples was calculated by comparing the sample OD to the standard curve.

**Statistical analysis**

Statistical analyses were performed using GraphPad Prism software (GraphPad Software, San Diego, CA, USA). Statistically significant differences were assessed by independent unpaired parametric 2-tailed Student’s *t* test for comparison between two groups. For experiments that contained more than two groups, ANOVA tests were used. All values were presented as mean ± SEM. Results were considered significant when the P value was <0.05.

**Supplementary Documents**

| **List of Reagents** | | | | |
| --- | --- | --- | --- | --- |
| **Antibody/Reagents** | **Color** | **Cat. #** | **Company** | **Species Reactivity** |
| **Viability Dye eFluor™ 780** |  | 65-0865-14 | eBioscience |  |
| **FBP1** | FITC | orb8080 | Biorbyt | Human |
| **FBP1** |  | PIMA536138 | Invitrogen | Human |
| **Anti-rabbit IgG** | Alexa-488 | A11008 | Invitrogen |  |
| **Anti-rabbit IgG** | Alexa-555 | A31572 | Invitrogen |  |
| **VDR** | PE | LS-C273294 | Life Span Biosciences | Human |
| **IgG1, κ Isotype Ctrl** | FITC | 400107 | Biolegend |  |
| **β-actin** |  | A-1978 | Sigma Aldrich |  |
| **DAPI** |  | D9542-1MG | Sigma Aldrich |  |
| **Midostaurin** (**MIDO**) |  | M1323-5MG | Sigma Aldrich |  |
| **Gilteritinib (GILT)** |  | S7754 | SELLECKCHEM |  |
| **1α,25-Dihydroxyvitamin D3** |  | H-089-1ML | Sigma Aldrich |  |
| **Lactate Assay Kit** |  | MAK064-1KT | Sigma Aldrich |  |

**Supplementary** **Table 1: List of Reagents used in this study**


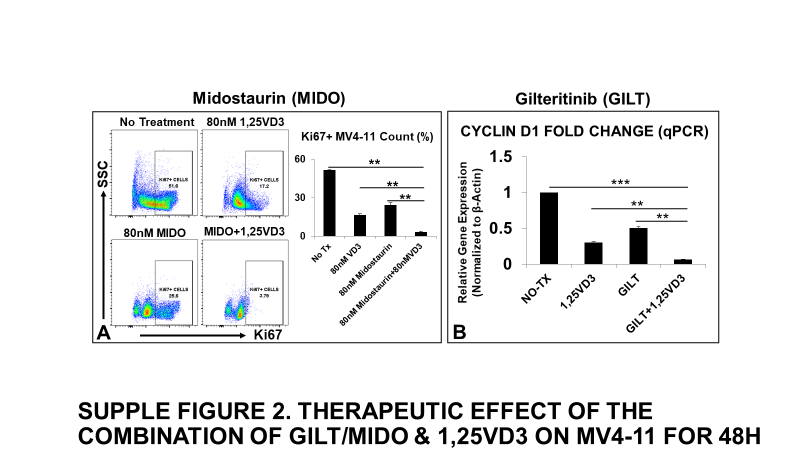


**Supplementary** **Figure 1: Therapeutic effect of the combination therapy of 80nM active 1,25 vitamin D (1,25VD3) and 80nM Tyrosine Kinase Inhibitors (TKIs) on MV4-11. *A)***: Midostaurin (MIDO), a 1^st^ generation TKI; ***B)***: Gilteritinib (GILT), a 2^nd^ generation TKI.

***A)*** Representative Flow Cytometry (FC) plots and FC percentage data of viable Ki67+ MV4-11 cells in different treatment groups with or without 80nM 1,25VD3 and/or 80nM MIDO;

***B)*** The MV4-11 cells from different treatment groups with or without 80nM 1,25VD3 and/or 80nM GILT were collected for RNA isolation and gene expressions were analyzed by qPCR. Data show mRNA expressions of the gene encoding CYCLIN D1;

*P<0.05, **P<0.01, ***P<0.005, n=5.


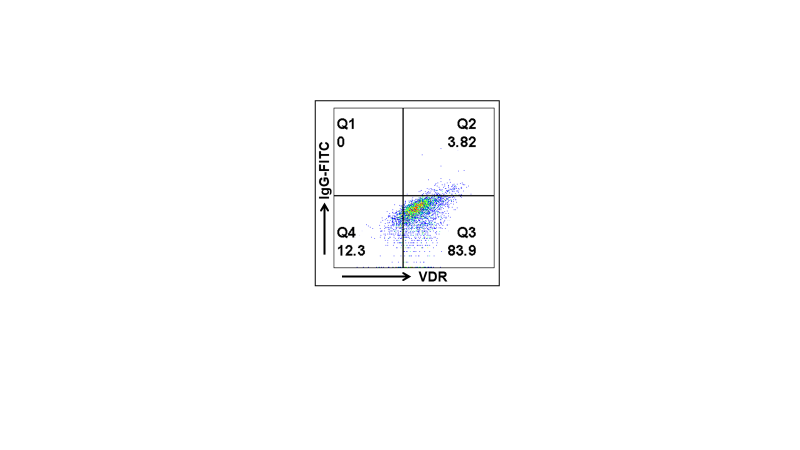


**Supplementary** **Figure 2: Isotype control.**

MV4-11 cells were stained with a mouse IgG monoclonal antibody conjugated to FITC and anti-human Vitamin D receptor (VDR)-PE antibody.


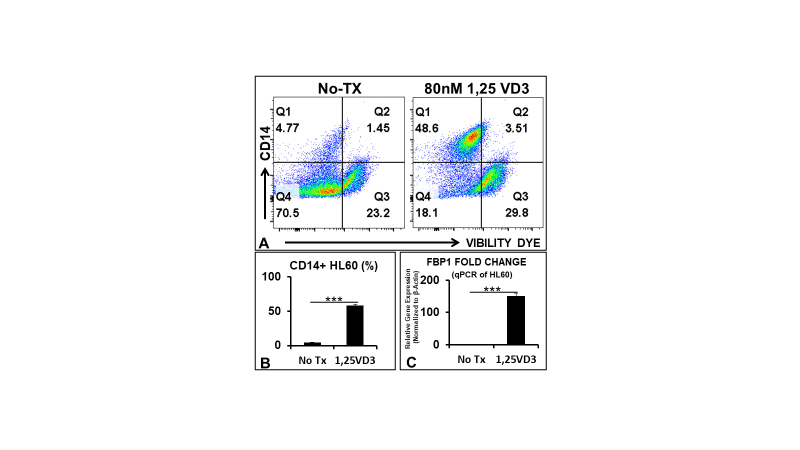


**Supplementary** **Figure 3: Therapeutic effect of 80nM active 1,25 vitamin D (1,25VD3) on HL60, a promyeloblasts derived from an acute promyelocytic leukemia (APL) patient (based on ATCC information).**

***A)*** Representative FC plots of viable CD14+ HL60 in different treatment groups;

***B)*** Cumulative FC percentage data of viable CD14+ HL60 cells;

***C)*** The HL60 cells treated with or without 1,25VD3 were collected for RNA isolation and gene expressions were analyzed by qPCR. Data show mRNA expressions of the FBP1 gene;

***P<0.005, n=3.

**References**

1. Xu, Y., et al., *A novel vitamin D gene therapy for acute myeloid leukemia.* Transl Oncol, 2020. **13**(12): p. 100869.

2. Corchete, L.A., et al., *Systematic comparison and assessment of RNA-seq procedures for gene expression quantitative analysis.* Sci Rep, 2020. **10**(1): p. 19737.

3. Cao, H., et al., *Discovery of proangiogenic CD44+mesenchymal cancer stem cells in an acute myeloid leukemia patient's bone marrow.* J Hematol Oncol, 2020. **13**(1): p. 63.

4. Xu, Y., et al., *Neurogenesis in the ependymal layer of the adult rat 3rd ventricle.* Exp Neurol, 2005. **192**(2): p. 251-64.
